# Immunometabolic Reprogramming of Fibroblastic Reticular Cells in the Tumor Immune Microenvironment

**DOI:** 10.1155/bmri/1172509

**Published:** 2026-07-10

**Authors:** Tian Tian, Huimin Liu, Peiyan Liu, Shumin Ye, Yudi Wu, Jun Zhou

**Affiliations:** ^1^ Department of Pulmonary and Critical Care Medicine, Changzhou Medical Center, Nanjing Medical University, Changzhou City, China, njmu.edu.cn; ^2^ Department of Pulmonary and Critical Care Medicine, The First People′s Hospital of Changzhou, Changzhou City, China

**Keywords:** fibroblastic reticular cell, immunometabolic reprogramming, multiple cancer types, tumor immune microenvironment

## Abstract

Fibroblastic reticular cells (FRCs), as core stromal cells in secondary lymphoid tissues and the tumor immune microenvironment (TIME), undergo significant immunometabolic reprogramming, which regulates antitumor immune responses. This structured narrative review summarizes the immunometabolic reprogramming of FRCs across various cancers, emphasizing glucose metabolism, lipid remodeling, and amino acid metabolism in lung cancer, breast cancer, gastric cancer, lymphoma, head and neck tumors, and melanoma. Under hypoxia, nutrient stress, and inflammatory stimulation, FRCs enhance glycolysis, alter fatty acid synthesis/oxidation, and disrupt amino acid metabolism, leading to immunosuppressive metabolite secretion, cytokine profile changes, and the formation of immune niches. Understanding these cancer‐specific molecular mechanisms can inform targeted immunometabolic therapies.

## 1. Introduction

The advent of cancer immunotherapy, particularly the successful clinical application of immune checkpoint inhibitors [1–3], has revolutionized the treatment of multiple advanced malignancies [4]. However, the overall efficacy is limited, and the majority of patients experience primary or secondary resistance [5–7], which remain significant challenges in clinical practice. Essentially, tumors not only establish an immunosuppressive microenvironment at the primary site but also hinder the initiation of antitumor immune responses at the systemic level. The key site for this process is the tumor‐draining lymph nodes (TdLNs) [8], which act as the origin and regulatory center of adaptive immune responses.

TdLNs [9–12] are the initial sites where naive T cells recognize tumor antigens, become activated, and differentiate into effector T cells. The efficiency and quality of this process directly determine the quantity and function of T cells that subsequently infiltrate the tumor. In the T cell area of TdLNs, there exists a highly specialized stromal cell—fibroblastic reticular cell (FRC) [13–16]—which forms a fine three‐dimensional reticular structure. Traditionally, it was believed to be a physical scaffold for the migration and interaction of T cells and dendritic cells. However, research over the past decade has completely revolutionized this understanding: FRCs are active and indispensable regulators of the microenvironment in B and T‐cell areas. Besides forming a reticular scaffold that guides the migration of immune cells, under steady‐state conditions, they support the survival and homeostasis of naive T cells and memory T cells by secreting CCL19 [17], CCL21 [18], CXCL12 [19], and IL‐7 [20–22] and providing metabolic substrates such as lactate [23]. During the tumor development and progression, the dynamic interaction between tumor cells and the immune microenvironment drives significant functional remodeling of FRCs. Metabolic reprogramming, as the core regulatory mechanism of cellular functional transformation, has become a key entry point for understanding the role of FRCs in tumor immunity.

The tumor immune microenvironment (TIME) of different cancer types exhibits significant heterogeneity. For instance, the degree of hypoxia in lung cancer (LC), the level of interstitial fibrosis in breast cancer, and the characteristics of lymphoid tissue remodeling in lymphoma show obvious differences. This may lead to cancer‐specific patterns of metabolic reprogramming in FRCs residing in the cancer stroma [24, 25]. Currently, studies on the metabolic reprogramming of FRCs have initially revealed some regulatory mechanisms in LC, breast cancer, and other cancers [26]. However, systematic analyses of the heterogeneity of FRCs′ metabolic reprogramming, core regulatory pathways, and immune regulatory effects in different cancers are still scarce. LC, as the most prevalent and lethal malignant tumor worldwide, has a relatively low response rate to immunotherapy. Recent studies [27, 28] have confirmed that FRCs construct protective T‐cell niches in the LC immune microenvironment, and their metabolic status may be a key factor influencing the efficacy of immunotherapy. Therefore, this article focuses on LC and combines multiple other cancer types to systematically review the molecular mechanisms of FRCs′ metabolic reprogramming, cancer‐specific features, and their regulatory roles in tumor immunity, aiming to provide theoretical support for the development of novel cancer immunotherapy strategies.

## 2. Methodology

This review followed a structured narrative review approach. Literature searches were conducted systematically using PubMed, Web of Science, and Scopus databases from inception to January 2026. The search strategy combined terms including “fibroblastic reticular cells,” “tumor immune microenvironment,” “ immunometabolic reprogramming,” and specific cancer types (lung, breast, gastric, lymphoma, head and neck, and melanoma). Inclusion criteria were original research and reviews discussing FRC metabolic pathways, molecular mediators, and immune effects in cancer. Exclusion criteria included studies lacking primary data on FRCs or those not addressing metabolic pathways. Article selection was performed independently by two reviewers, and discrepancies were resolved through discussion.

## 3. The Core Mechanism of FRCs′ Metabolic Reprogramming

### 3.1. Glucose Metabolism

Under the influence of the TIME, the energy metabolism pathways of FRCs undergo significant changes. Under normal circumstances, FRCs mainly rely on oxidative phosphorylation (OXPHOS) to produce ATP. However, in response to IL‐17 [23, 29, 30] signaling stimulation, FRCs transition from a resting state to a highly metabolically active state, characterized by enhanced glycolytic activity and increased mitochondrial respiratory levels. This metabolic remodeling process provides the necessary energy and biosynthetic precursors for the immunomodulatory function of FRCs. Activated FRCs further act as the “metabolic support units” for T cells [31] by releasing metabolic substrates such as lactic acid (LA) and amino acids and secreting signaling molecules such as IL‐6, systematically remodeling the metabolic pathways of T cells, including promoting their glycolytic and OXPHOS levels, thereby supporting the differentiation of T cells into effector cells or memory cells. This metabolic transformation not only meets the energy requirements of FRCs in the inflammatory environment but also actively regulates the functional state of surrounding immune cells by generating a large amount of metabolic products such as LA.

As the final product of glycolysis, LA plays multiple regulatory roles in the immunosuppressive process mediated by FRCs [32]. Firstly, after FRCs take up LA, it is mainly converted into pyruvate, which then generates acetyl carnitine and *α*‐ketoglutarate/glutamate, instead of being powered by the tricarboxylic acid cycle. This metabolic pathway simultaneously reduces the synthesis of glutathione and weakens the antioxidant capacity of FRCs. Secondly, the LA‐driven reprogramming process of FRCs does not rely on the hypoxia‐inducible factor 1*α* signaling pathway but instead, through a mechanism similar to that of nigericin, directly lowers the intracellular pH, triggering overall metabolic changes, thereby interfering with the metabolic reprogramming and effector functions of T cells [33]. Moreover, LA can directly promote the upregulation of Podoplanin and Thy1 expression in FRCs, while inhibiting the expression of IL‐7, indicating that LA is a key molecule promoting the transformation of FRCs to the “promoting tumor activation state.”

### 3.2. Amino Acid Metabolism

The reconfiguration of amino acid metabolism is another significant feature of the metabolic reprogramming of FRCs in the TIME. Among them, the research on the tryptophan‐kynurenine pathway is the most in‐depth. Under the stimulation of inflammatory factors, FRCs highly express indoleamine 2,3‐dioxygenase (IDO) [31, 34], which is the rate‐limiting enzyme for the catabolism of tryptophan along the kynurenine pathway. The activation of IDO leads to local depletion of tryptophan and accumulation of kynurenine, which in turn inhibits T‐cell function through multiple mechanisms.

The depletion of tryptophan can activate the GCN2 kinase [35] which globally inhibits protein synthesis through eukaryotic translation initiation factors 2*α* [36], leading to T‐cell cycle arrest [37]. At the same time, the accumulated kynurenine can activate the aryl hydrocarbon receptor (AHR) [38, 39] promoting the differentiation of regulatory T cells and inhibiting the function of effector T cells. It is noteworthy that the activation of AHR can also positively feedback upregulate the expression of IDO1, forming a self‐amplifying immunosuppressive cycle.

In addition to tryptophan metabolism, arginine metabolism also plays a significant role in the immune regulation mediated by FRCs. In an inflammatory environment, FRCs can express arginase I and inducible nitric oxide synthase (iNOS) [40]. These two enzymes compete for the substrate L‐arginine. The nitric oxide (NO) [41, 42] produced by iNOS can inhibit T‐cell function through various pathways such as inhibiting mitochondrial respiratory chain complexes and interfering with T‐cell receptor signal transduction.

### 3.3. Lipid Metabolism

In addition to glucose and amino acid metabolism, lipid metabolic remodeling represents an important but relatively underexplored component of FRC‐mediated immune regulation. Lipid metabolism in FRCs should not be viewed only as an energetic process involving fatty‐acid synthesis and oxidation; rather, it also generates bioactive lipid mediators that directly regulate immune‐cell activation, migration, and tolerance. During inflammatory or tumor‐associated stimulation, altered fatty‐acid synthesis may support FRC proliferation, membrane remodeling, and extracellular matrix reorganization, whereas changes in fatty‐acid oxidation may influence mitochondrial OXPHOS [43] and the capacity of FRCs to maintain lymphoid tissue structure and immune‐cell niches.

A key lipid‐associated pathway in FRCs is the cyclooxygenase‐2/prostaglandin E2 (COX‐2/PGE2) [44–46] axis. Lymphoid tissue FRCs constitutively express high levels of COX‐2 and produce PGE2, which can increase the activation threshold of T cells and attenuate excessive T‐cell responses. Mechanistically, PGE2 acts through EP2 and EP4 receptors, leading to cAMP‐dependent signaling that suppresses T‐cell activation and may also impair dendritic‐cell maturation and antigen‐presenting function. In the TdLN, persistent tumor antigen exposure and inflammatory stimulation may further amplify this PGE2‐dependent immunoregulatory loop, thereby weakening effective antitumor T‐cell priming and contributing to immune tolerance.

FRC‐derived lipid‐related signals may also regulate B‐cell metabolism. Recent evidence shows that spleen FRC‐derived acetylcholine promotes lipid influx, lipid metabolism, and mitochondrial OXPHOS in B cells, thereby driving autoreactive B‐cell responses. Although this mechanism has been mainly characterized in autoimmune settings, it suggests that FRCs can regulate immune responses not only by remodeling their own lipid metabolism but also by reshaping the lipid metabolic state of neighboring lymphocytes [47]. In tumor‐draining lymphoid tissues, such FRC–B‐cell metabolic crosstalk may influence antibody responses, tertiary lymphoid structure function, and local immune tolerance.

Functionally, lipid metabolic reprogramming of FRCs may contribute to tumor immune escape through several mechanisms: inhibition of dendritic‐cell maturation, elevation of T‐cell activation thresholds, suppression of effector T‐cell function, promotion of tolerance‐associated immune responses, and support of lymph‐node stromal remodeling. These findings also suggest that targeting the COX‐2/PGE2/EP2/EP4 [48] pathway, or modulating lipid metabolic dependencies of FRCs, may represent a potential strategy to improve antitumor immunity and overcome resistance to immunotherapy. However, direct evidence regarding cancer‐specific lipid metabolic programs in tumor‐associated FRCs remains limited, and future studies using spatial metabolomics, lipidomics, and FRC‐specific genetic models are needed to clarify these mechanisms.

**Figure 1:**
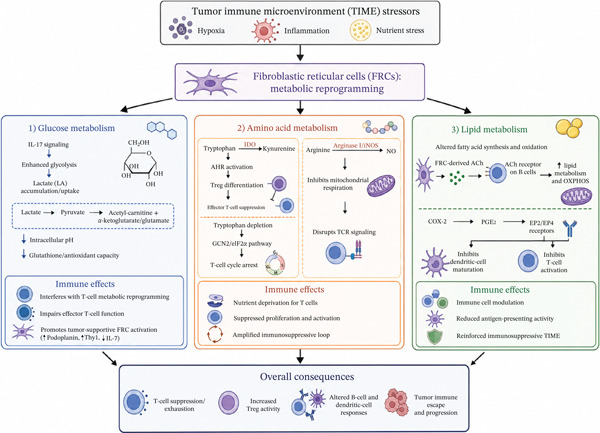
Schematic diagram of the major metabolic pathways involved in fibroblastic reticular cell (FRC) reprogramming in the tumor immune microenvironment.

To improve clarity, a schematic summary of the major glucose, amino acid, and lipid metabolic pathways involved in FRC reprogramming and their immune consequences is provided in Figure 1.

Under hypoxia, inflammation, and nutrient stress, FRCs undergo metabolic reprogramming involving enhanced glycolysis, dysregulated amino acid metabolism, and altered lipid metabolism. Key mediators include lactate, IDO, kynurenine, arginase I, iNOS, NO, COX‐2, and PGE2. These changes reshape T‐cell, B‐cell, and dendritic‐cell responses, thereby contributing to immunosuppression and tumor immune escape.

## 4. LC: Metabolic Reprogramming of FRCs and the Focus of Immune Therapy Resistance

LC (Table 1), especially non‐small cell lung cancer (NSCLC), has a TIME with significant hypoxic characteristics and a complex interstitial–immune cell interaction network. This is a paradigm for studying the metabolic reprogramming of FRCs and its association with clinical outcomes.

**Table 1:** Comparison of metabolic reprogramming of fibroblastic reticular cells across different cancer types.

Cancer type	Primary metabolic pathway	Key molecules	Immune effects/consequences
Lung cancer (NSCLC)	Metabolic niche construction (minimal self‐reprogramming)	CCL19, IL‐7, and POSTN	Supports T‐cell survival and activation; reduces exhaustion; niche formation influences immune therapy response
Breast cancer (including TNBC)	Mitochondrial OXPHOS reprogramming	PD‐L1, iNOS, IL‐7, and CCL21	Increases Tregs, inhibits CD4/CD8 T‐cell proliferation, creates immunosuppressive microenvironment; impaired T‐cell migration
Gastric cancer	Inflammatory chemokine signaling and TGF‐*β*1–mediated activation	CCL2, CXCL1, ITGB1, and TNS4	Recruits myeloid cells; amplifies local inflammation; promotes lymph node metastasis
Head and neck (HNSCC/OSCC)	JAK1‐STAT1 activation and loss of chemokines	IFNGR1, PD‐L1, and CCL19/CCL21	Induces CD8^+^ T‐cell exhaustion; reduces immune cell infiltration; promotes metastasis
Melanoma	IL‐1*α*/IL‐1*β*–mediated activation	FAP	Decreases FRC contractility; enhances tumor adhesion and proliferation; supports immunosuppression
Lymphoma (DLBCL and FL)	Structural remodeling and immunosuppressive metabolite secretion	FAP, PD‐L1, PD‐L2, IDO, and PGE2	Inhibits CD8^+^ T‐cell cytotoxicity; supports tumor survival; promotes Tfh proliferation and IL‐4/CXCL12 feedback loop

In the TIME of LC [27, 49], FRCs expressing CCL19 originate from mesenchymal progenitor cells and adventitial fibroblasts in the vascular walls of lung tissue and differentiate into two functionally specialized subpopulations: perivascular reticular cells and T‐cell zone reticular cells. These FRCs form a three‐dimensional reticular structure by interconnecting, creating tertiary lymphoid structures at the tumor margin and extending into the tumor parenchyma through “T‐cell conduits.” They establish a CCL19 chemokine gradient and directly physically interact with CD8^+^ T [50, 51] cells, thereby guiding T cells to migrate towards the tumor site and promoting their activation, clonal expansion, and complete effector function differentiation in the lymphoid structures and perivascular niches. Additionally, FRCs maintain the metabolic adaptability and functional persistence of T cells and inhibit their exhaustion state by providing key molecular signals such as IL‐7 and POSTN, ultimately establishing a local protective immune environment that effectively suppresses tumor progression.

Although its core mechanism lies in providing structural and signaling support rather than undergoing significant metabolic reprogramming itself, the niche established by FRCs essentially regulates the metabolic state and immune function of infiltrating T cells, becoming an indispensable regulatory hub in the TIME of LC. Experiments have shown [28] that specific ablation of CCL19^+^ FRCs disrupts the integrity of this niche, leading to decreased function and increased exhaustion of tumor‐infiltrating T cells, thereby accelerating tumor growth. These findings not only reveal that FRCs are structural and signaling supports in the LC TIME but also provide new therapeutic strategies for enhancing antitumor immunity by targeting or reprogramming tumor stromal cells in the future.

## 5. The Similarities and Differences in Metabolic Reprogramming of Fibroblasts in Other Cancer Types

Although the core mechanism is shared, the metabolic reprogramming of FRCs in different cancer types exhibits interesting heterogeneity due to their unique etiology, anatomical location, and tumor biology.

### 5.1. Breast Cancer (Table 1)

The research [52] found that under breast cancer induction, the FRCs in TDLNs undergo mitochondrial OXPHOS metabolic reprogramming, accompanied by the downregulation of T‐cell anti‐inflammatory gene expression, an increase in Treg quantity, and a reduction in marginal zone B cells, jointly constructing an immunosuppressive microenvironment. In more aggressive subtypes, triple‐negative breast cancer [40, 53] due to its higher immunogenicity, may induce more severe metabolic inhibition. During this process, the monocytes recruited by FRCs exhibit high expression of PD‐L1 and iNOS, and through iNOS‐mediated NO release, inhibit the proliferation of CD4^+^ and CD8^+^ T cells, while downregulating T‐cell migration‐related genes, thereby weakening their immune killing function. This series of changes form an immunosuppressive microenvironment that creates favorable conditions for tumor cell escape and promotes the distant metastasis of tumor cells in TDLNs.

Further studies have also revealed that in the lymph nodes of patients with invasive breast cancer [54], FRCs often exhibit reduced or even absent expression of CCL21, along with decreased expression of heparan sulfate, resulting in the inability of CCL21 to effectively bind to the cell surface and thereby affecting the migration ability of lymphocytes. These changes are often associated with features such as dilation of high endothelial venules, aggregation of lymphocytes around blood vessels, and immunosuppression. Abnormal function of FRCs is likely to enhance the immune tolerance state within the lymph nodes, thereby promoting tumor immune escape. Relevant studies suggest that the dysfunction of FRCs may not only reflect the invasive characteristics of the tumor but also serve as a biomarker for predicting disease progression.

### 5.2. Gastric Cancer

In the TIME of gastric cancer [55] (Table 1), malnutrition and the enrichment of inflammatory factors are two typical characteristics. Studies have found [56] that FRCs secrete chemokines such as CCL2 and CXCL1, which recruit myeloid cells and amplify the local inflammatory response. Additionally, related research [57] further reveals that on the surface of FRCs, integrin beta 1 (ITGB1) can directly interact with Tensin 4 (TNS4) to enhance the activation of the downstream SMAD2/3 and AKT signaling pathways of transforming growth factor *β*1 (TGF‐*β*1), thereby driving the functional activation of FRCs. This mechanism is closely related to the significant increase in the lymph node metastasis rate of gastric cancer.

### 5.3. Head and Neck Squamous Cell Carcinoma (HNSCC)

In the lymph nodes where micrometastasis occurs in patients with HNSCC (Table 1), the proportion of FRCs significantly increases. Studies have shown [58] that interferon gamma receptor 1 (IFNGR1) can be engulfed by FRCs, and through the activation of the JAK1‐STAT1 signaling pathway, it induces an increase in PD‐L1 expression on the surface of FRCs, thereby exacerbating the depletion of CD8^+^ T cells [59] and promoting the tumor metastasis process.

As an important subtype of HNSCC, cervical lymph node metastasis of oral squamous cell carcinoma (OSCC) is a key factor influencing the prognosis of patients. Studies have found [60] that under the stimulation of the tumor microenvironment, FRCs can transform into Type 2 cancer‐associated myofibroblasts, accompanied by the loss of immune regulatory functions—manifested as the downregulation of chemokines CCL19/CCL21 and the enhancement of extracellular matrix remodeling ability. This transformation process not only weakens the local immune response but also reduces the infiltration of immune cells, jointly constructing a tumor microenvironment with dual promoting metastasis properties.

### 5.4. Melanoma

Lymphatic metastasis of melanoma (Table 1) is a key factor contributing to poor prognosis in patients. Studies have shown [61] that after melanoma infiltration, FRCs undergo critical functional reprogramming. The expression of fibroblast activation protein (FAP) significantly increases, while their contractile ability decreases. These changes are closely related to the enhanced invasiveness of the tumor and the intensification of immunosuppression. Further research [62] reveals that the core regulatory factors IL‐1*α*/IL‐1*β* secreted by dedifferentiated melanoma cells can significantly weaken the contractile ability of FRCs, promote their proliferation and activation, provide adhesion and growth signals for tumor cells, and thereby create a microenvironment conducive to tumor metastasis.

### 5.5. Lymphoma

Lymphoma (Table 1) directly originates from the lymphatic system and thus has a more direct and complex interaction with FRCs. Studies have shown [63] that in diffuse large B‐cell lymphoma (DLBCL), FRCs undergo abnormal structural remodeling and transcriptional reprogramming under the influence of DLBCL cells, manifesting as high expression of FAP and upregulation of co‐inhibitory ligands such as PD‐L1 and PD‐L2. These changes inhibit the migration and cytotoxicity of CD8^+^ tumor‐infiltrating lymphocytes and CAR‐T cells, thereby weakening the antitumor immune response.

In the TIME of follicular lymphoma (FL) [64], FRCs act as the core hub that supports tumor growth and regulates immunity. They not only provide structural support and directional guidance for lymphocytes by secreting chemokines but also actively shape an immunosuppressive environment by expressing molecules such as IDO and PGE2 to inhibit T‐cell function. Moreover, FRCs promote the proliferation and IL‐4 secretion of follicular helper T cells through Notch and ICAM‐1/LFA‐1 signaling pathways, forming a positive feedback loop of IL‐4/CXCL12, directly supporting the survival, migration, and adhesion of FL B cells. At the same time, FRCs participate in extracellular matrix remodeling and angiogenesis. The network structure formed by FRCs and follicular dendritic cells is closely related to the prognosis of patients. Therefore, FRCs have become an important potential therapeutic target.

## 6. Summary and Outlook

FRCs are key regulatory cells in the TIME. Their immunometabolic reprogramming profoundly influences the intensity and direction of tumor immune responses by reshaping metabolic pathways such as glucose metabolism, lipid metabolism, and amino acid metabolism. The immunometabolic reprogramming of FRCs varies significantly across different cancers. In LC, the metabolic reprogramming of FRCs in the mediastinal lymph nodes is an important factor leading to impaired T‐cell function and resistance to immune checkpoint inhibitors. Its expression level has prognostic and predictive value and is a potential combined treatment target. This mechanism has been confirmed in various cancers such as breast cancer, gastric cancer, head and neck tumors, melanoma, and lymphoma. However, the strength of the driving signals, the dominant metabolic pathways, and the impact on treatment responses vary depending on the cancer type, reflecting the complexity of tumor biology and anatomical location. These metabolic reprogramming patterns form an immune‐inhibitory network by secreting immunosuppressive metabolic products, consuming essential nutrients for immune cells, and regulating cytokine secretion, promoting tumor immune escape.

Targeting the immunometabolic reprogramming of FRCs has become a new direction in cancer immunotherapy. Currently, inhibitors targeting glycolysis, amino acid metabolism, and upstream signaling pathways have shown excellent antitumor effects in preclinical studies, and some drugs have entered clinical trial stages. However, the metabolic plasticity, tissue specificity, and spatiotemporal heterogeneity of FRCs still pose challenges for clinical translation, and it is necessary to elucidate the molecular mechanisms of FRCs′ immunometabolic reprogramming through multi‐omics technologies and develop precise and multitargeted combined treatment regimens.

## 7. Critical Analysis and Limitations

While this review consolidates evidence on FRC metabolic reprogramming, variability in experimental models, patient heterogeneity, and limited longitudinal studies restrict generalizability. Many studies focus on single cancer types or in vitro models, which may not fully capture in vivo TIME complexity. Additionally, methodological differences in measuring metabolic fluxes and immune effects limit cross‐study comparisons. Future work should integrate multi‐omics analyses and standardized metabolic assays to clarify mechanistic pathways and therapeutic potential.

## 8. Conclusion

FRC immunometabolic reprogramming is a pivotal determinant of tumor immune landscapes. Enhanced glycolysis, lipid remodeling, and amino acid metabolism collectively shape immune suppression or activation across cancers. Recognizing methodological limitations and promoting transparent, reproducible research will improve translation of these insights into immunotherapy strategies.

**Nomenclature**
TdLN: tumor‐draining lymph node
FRC: fibroblastic reticular cell
LA: lactic acid
IDO: indoleamine 2,3‐dioxygenase
AHR: aryl hydrocarbon receptor
iNOS: inducible nitric oxide synthase
NO: nitric oxide
ACh: acetylcholine
TIME: tumor immune microenvironment
OXPHOS: oxidative phosphorylation
PGE2: prostaglandins E2
LC: lung cancer
NSCLC: non‐small cell lung cancer
TNBC: triple‐negative breast cancer
HNSCC: head and neck squamous cell carcinoma
IFNGR1: interferon gamma receptor 1
OSCC: oral squamous cell carcinoma
FAP: fibroblast activation protein
DLBCL: diffuse large B‐cell lymphoma
FL: follicular lymphoma

## Author Contributions

Tian Tian: conception and manuscript drafting; Huimin Liu and Peiyan Liu: literature screening and manuscript revision; Shumin Ye: manuscript revision; Jun Zhou: conception, supervision, final review. Tian Tian, Huimin Liu, and Peiyan Liu made equal contributions to this study and co‐first authors.

## Funding

This study was supported by Changzhou Municipal Science and Technology Bureau, 10.13039/501100007131, CE20235057.

## Consent

The authors have nothing to report.

## Conflicts of Interest

The authors declare no conflicts of interest.

## Data Availability

No datasets were generated or analyzed during this review.
